# Social dialogue triggers biobehavioral synchrony of partners' endocrine response via sex-specific, hormone-specific, attachment-specific mechanisms

**DOI:** 10.1038/s41598-021-91626-0

**Published:** 2021-06-14

**Authors:** Amir Djalovski, Sivan Kinreich, Orna Zagoory-Sharon, Ruth Feldman

**Affiliations:** 1 Center for Developmental Social Neuroscience, Interdisciplinary Center Herzliya, Herzliya, Israel; 2 Department of Psychology, Bar-Ilan University, Ramat Gan, Israel; 3 Department of Psychiatry, State University of New York Downstate Medical Center, Brooklyn, NY USA; 4 Child Study Center, Yale University, New Haven, CT USA

**Keywords:** Human behaviour, Social behaviour

## Abstract

Social contact is known to impact the partners' physiology and behavior but the mechanisms underpinning such inter-partner influences are far from clear. Guided by the biobehavioral synchrony conceptual frame, we examined how social dialogue shapes the partners' multi-system endocrine response as mediated by behavioral synchrony. To address sex-specific, hormone-specific, attachment-specific mechanisms, we recruited 82 man–woman pairs (N = 164 participants) in three attachment groups; long-term couples (n = 29), best friends (n = 26), and ingroup strangers (n = 27). We used salivary measures of oxytocin (OT), cortisol (CT), testosterone (T), and secretory immuglobolinA (s-IgA), biomarker of the immune system, before and after a 30-min social dialogue. Dialogue increased oxytocin and reduced cortisol and testosterone. Cross-person cross-hormone influences indicated that dialogue carries distinct effects on women and men as mediated by social behavior and attachment status. Men's baseline stress-related biomarkers showed both direct hormone-to-hormone associations and, via attachment status and behavioral synchrony, impacted women's post-dialogue biomarkers of stress, affiliation, and immunity. In contrast, women's baseline stress biomarkers linked with men's stress response only through the mediating role of behavioral synchrony. As to affiliation biomarkers, men's initial OT impacted women's OT response only through behavioral synchrony, whereas women's baseline OT was directly related to men's post-dialogue OT levels. Findings pinpoint the neuroendocrine advantage of social dialogue, suggest that women are more sensitive to signs of men's initial stress and social status, and describe behavior-based mechanisms by which human attachments create a coupled biology toward greater well-being and resilience.

## Introduction

Close relationships confer significant benefits to physical and mental health; buffer stress, foster optimism, generate productivity, and facilitate resilience^1,2^, whereas social isolation leads to depression, mental deterioration, illness, and, in extreme cases, death^3^. Still, the mechanisms by which relationships exert their effects are far from clear and require much further research. While earlier perspectives addressed the security^4^, sense of meaning^5^, or potential for growth^6^ afforded by intimate relationships, recent models search for their biological underpinnings, adopt an evolutionary perspective, and highlight the provisions embedded in affiliative bonds and group living for the survival and thriving of mammals^7^. In humans, hormonal correlates of positive or stressful relationships^8,9^, the neural systems implicated in romantic love and friendship^10^, and the soothing effects of a friend's touch^11^ have been described. Despite these discrete studies, a comprehensive assessment on how human attachments trigger a multisystem endocrine response, including markers of affiliation, stress, social dominance, and immunity as mediated by relationship quality, is lacking. Such integration across systems and relationships may provide a panoramic view for the effects of social contact on a broad-band endocrine response, charting mechanisms by which social partners influence each other's physiology and behavior and pinpointing how social dialogue differentially impacts the physiological response of women and men.

The human brain has undergone massive expansion across primate evolution through life amidst social affiliations: between couples, among friends, and within communities, and this enabled humans to build complex communicative systems by which they can fine-tune the physiological and behavioral response of social partners toward the execution of survival-related social goals^12^. The *biobehavioral synchrony* model^10,13^ proposes that coordinated social behavior within attachment bonds, beginning with the mother-infant bond, provides a template for the coordination of the partners' physiological systems; during moments of social synchrony partners synchronize their heart rhythms^14^, brain oscillations^15^, and hormonal secretion^16^. Such bio-behavioral mechanisms enable partners to up- or down-regulate each other's response through both direct hormone-to-hormone influences as well as mediated effects via synchronous behavior, impacting the partner's hormones by means of behavioral attunement.

In the current study, we tested the effects of social dialogue between man–woman pairs (N = 164 participants, 82 pairs) in three groups: (1) *Couples-* long-term romantic couples within a committed relationship and at least 1 year of cohabitation (n = 29); (2) *Friends*- close friends who considered each among their top five friends^12^ and their familiarity period matched the couples' (n = 26); and (3) *Strangers* demographically-matched unfamiliar group members (n = 27). We tested the effects of a naturalistic dialogue on four salivary biomarkers known to play a key role in sociality and well-being: oxytocin (OT), cortisol (CT), testosterone (T), and secretory immuglobolin A (s-IgA), a first-line mucosal barrier considered a biomarker of the immune system^17^, and examined how men and women uniquely impact their partner's salivary endocrine response both directly and through the coordination of social behavior.

The four hormones, particularly OT, CT, and T, not only facilitate and regulate social life by sustaining parental care, pair-bonding, social hierarchies, stress management, and group living across mammalian species, but evidence points to their complex inter-relationships^18^. For instance, OT has a modulatory function on both CT^19^ and T^20^; CT and T co-regulate each other's effects during intense or stressful tasks^21^; OT plays a pivotal role in immunity^22^; and hypothalamic–pituitary–adrenal (HPA)-axis over-activity, whose end product is CT, may have negative effects on the immune system^23,24^.

While these four hormones have not yet been integrated into a single study, assessing their cross-hormone mutual influences may expand our knowledge on the biological basis of human sociality, particularly as these systems maintain ongoing crosstalk. The OT system has widespread effects on body and brain^25^, underpins sociality and communication^26^, has anxiolytic, stress-reducing functions^27^, increases its activity following social contact^28^, and functions as an integrative interface for the effects of multiple hormones on health. OT plays a key role in adult attachments; it mediates pair-bond formation through connectivity with dopamine neurons in striatum^29^, increases during periods of falling in love^30^, and is associated with the degree of investment, satisfaction, and attunement in the couple relationship^31^.

In addition to its direct impact on human social communication and affiliation, OT interacts with CT, T, and immune biomarkers to improve well-being as mediated by behavioral synchrony. During periods of bond formation, links between OT and the immune system increase as a function of behavioral synchrony^32^; OT administration reduces HPA-axis activity in couples^33^; intranasal OT effects on fathers' T is modulated by father-child social synchrony^34^; and the co-regulatory influences of OT and s-IgA on adolescents' well-being are mediated by synchronous dialogue^35^. T, which is implicated in social dominance, competition, and aggression, particularly in males, is especially sensitive to attachment status and decreases in men within a committed partner relationship^36^ as well as in involved fathers^31^. Finally, among new lovers, OT was found to have a direct partner effect; individuals whose partner had high OT showed more behavioral empathy, pointing to the mutual influences among partners' hormones and behavior particularly with regards to the effects of OT^16^. Still, no study to date examined cross-hormonal influences between social partners as mediated by attachment status and social behavior or tested the cross-sex effects of women's hormonal profile on men and vice versa.

The goal of the current study was to describe how humans create a coupled biology through social dialogue and how processes of biobehavioral synchrony, by which partners impact each other's hormones through coordinated social behavior, are mediated by sex and attachment status and express differently in the various endocrine systems. Our overall hypothesis was that social dialogue would trigger a complex net of cross-person, cross-hormone influences between partners so that the baseline hormonal profile of one partner would impact the endocrine response of the other both directly and through the mediating role of behavioral synchrony (Hypothesis 1). We further expected that the effects of a man's baseline hormones on the woman's hormonal response would differ from that of the woman's initial profile on the man's endocrine reactivity, regardless of relationship status and behavioral attunement (Hypothesis 2). Finally, we expected that affiliative partners (couples, close friends) would exhibit greater behavioral synchrony and exert greater impact on each other's endocrine response as compared to strangers (Hypothesis 3).

## Results

We first tested for demographic differences between groups and found no differences in demographic factors (*p* > 0.05; see Table 1).

**Table 1:** Demographic table.

Variable	Couples (N = 29)		Friends (N = 26)		Strangers (N = 27)		dfb	dfw	F/t	*P*-value
	Mean	SD	Mean	SD	Mean	SD				
Age	25.67	5.75	25.17	4.14	25.04	3.06	2	161	0.27	0.77
Education	13.79	1.67	13.25	2.45	13.39	1.62	2	161	0.96	0.39
Time together/time friends	3.83	2.85	4.10	2.39	–		0.31	0.76		
BDI Score	4.23	4.06	5.26	7.02	4.56	4.40	2	161	0.41	0.66

Age, education, and time together were measured in years, *BDI* Beck Depression Inventory.

Prior to presenting our data, it is important to emphasize that our results do not imply causality, only associations, and terms "effects", "impact", and "influences" used here describes statistical, not causal effects.

Following, we examined group differences in *behavioral synchrony* and found significant group effect (*F*_*(2, 79)*_ = 44.47, *p* < 0.01, *η*_*p*_^*2*^ = 0.53): *Couples* (M = 2.28, SD = 0.27) and *Friends* (M = 2.19, SD = 0.11) scored significantly higher than *Strangers* (M = 1.85, SD = 0.17; *p* < 0.01; Bonferroni corrected).

**Figure 1:**
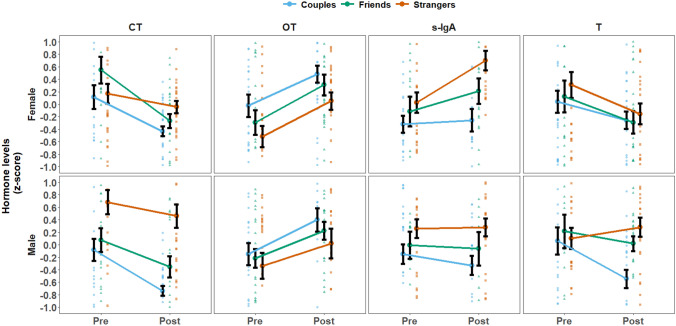
Group differences in CT, OT, s-IgA, and T. Groups are marked with shape and color (Couples*—*blue circle, Friends*—*green triangle, Strangers*—*orange square). Mean values are marked with bold square. Shaded dots are single participants values. Error bars reflects standard error from the mean.

Results of the mixed-models on the effects of social dialogue on women's and men's endocrine response in each hormone according to attachment group appear in Fig. 1. Significant overall main effect for time emerged for OT (*F*_*(1, 79)*_ = 48.86, *p* < 0.01), CT (*F*_*(1, 79)*_ = 27.49, *p* < 0.01), and T (*F*_*(1, 79)*_ = 13.86, *p* < 0.01); Social dialogue increased OT levels (M = − 0.25, SD = 0.94 vs. M = 0.25, SD = 0.9; respectively, *p* < 0.01), whereas CT (M = 0.25, SD = 1.01) and T (M = 0.14, SD = 1.12) were reduced following the dialogue (M = − 0.24, SD = 0.75, M = − 0.16, SD = 0.82; respectively, *p* < 0.01). These findings indicate that social dialogue increases biomarkers of affiliation and calm (OT) and decreasing markers of stress (CT) and competition (T).

Group effects were found for CT (*F*_*(2, 79)*_ = 17.99, *p* < 0.01), s-IgA (*F*_*(1, 79)*_ = 7.82, *p* < 0.01), T (*F*_*(1, 79)*_ = 4.17, *p* < 0.05), and OT (*F*_*(2, 79)*_ = 3.13, *p* < 0.05) with *Couples* exhibiting lower CT (M = − 0.28, SD = 0.82; *p* < 0.01), lower s-IgA (M = − 0.26, SD = 0.85; *p* < 0.01), and lower T (M = − 0.17, SD = 0.95; *p* < 0.05) but higher OT (M = 0.18, SD = 0.94; *p* < 0.05) compared to *Strangers* (M = 0.32, SD = 0.88; M = 0.32, SD = 0.82; M = 0.14, SD = 0.92, and M = − 0.2, SD = 1.01, respectively). In addition, significant differences in CT were also found between *Friends* and *Strangers* (M = − 0.02, SD = 0.95; *p* < 0.01).

Interaction effects were found for group and sex in CT (*F*_*(2, 79)*_ = 7.51, *p* < 0.05), for time and sex in s-IgA (*F*_*(1, 79)*_ = 4.94, *p* < 0.05; no significant simple effect was found), and for time-group-sex in T (*F*_*(2, 79)*_ = 3.58, *p* < 0.05). Among men, those in the *Strangers* group had significantly higher CT (M = 0.57, SD = 0.99; *p* < 0.01) compared to those in the *Couples* (M = − 0.41, SD = 0.8) or *Friends* (M = − 0.14, SD = 0.8) groups. Finally, post-dialogue CT among men in the *Couples* group (M = − 0.74, SD = 0.41), were lower than those in the *Friends* (M = − 0.36, SD = 0.87) and *Stranger* (M = 0.46, SD = 0.97) groups. These findings indicate that whereas the effect of social dialogue on increasing OT was ubiquitous across groups and sexes, the effects on reducing CT and T were more nuanced and, among men, depended on attachment status with the female partner.

Prior to computing the models, we assessed Pearson's correlations between hormones over time (see Table S1). Baseline and post-dialogue assessments within the same biomarker showed medium-to-high correlations, attesting to the intra-individual stability of these biomarkers and their test–retest reliability. Cross-sex correlations within the same biomarker emerged between baseline men's CT and post-dialogue women's CT and baseline women's OT and post-dialogue men's OT. Cross-hormone-cross-sex correlations were found between women's baseline s-IgA and men's post-dialogue CT, baseline men's CT and pre- and post-dialogue women's T, between men's baseline T and women's post-dialogue OT, and between men's baseline OT and women's post-dialogue T, highlighting the complex net of cross-sex, cross-time, and cross-hormones associations. It is important to emphasize that a large number of correlations were computed and these results should therefore be taken with caution and are only mentioned as background information to the following path analyses.

Finally, two path analyses models were computed to test the effects of one partner's baseline hormonal profile on the post-dialogue response of the interacting partner as mediated by behavioral synchrony and group. For a parsimonious model and since few differences were found between *Couples* and *Friends*, we combined the two groups into an *Affiliated* group (coded as 1) that was measured in comparison with the *Strangers (*coded as 0).

**Figure 2:**
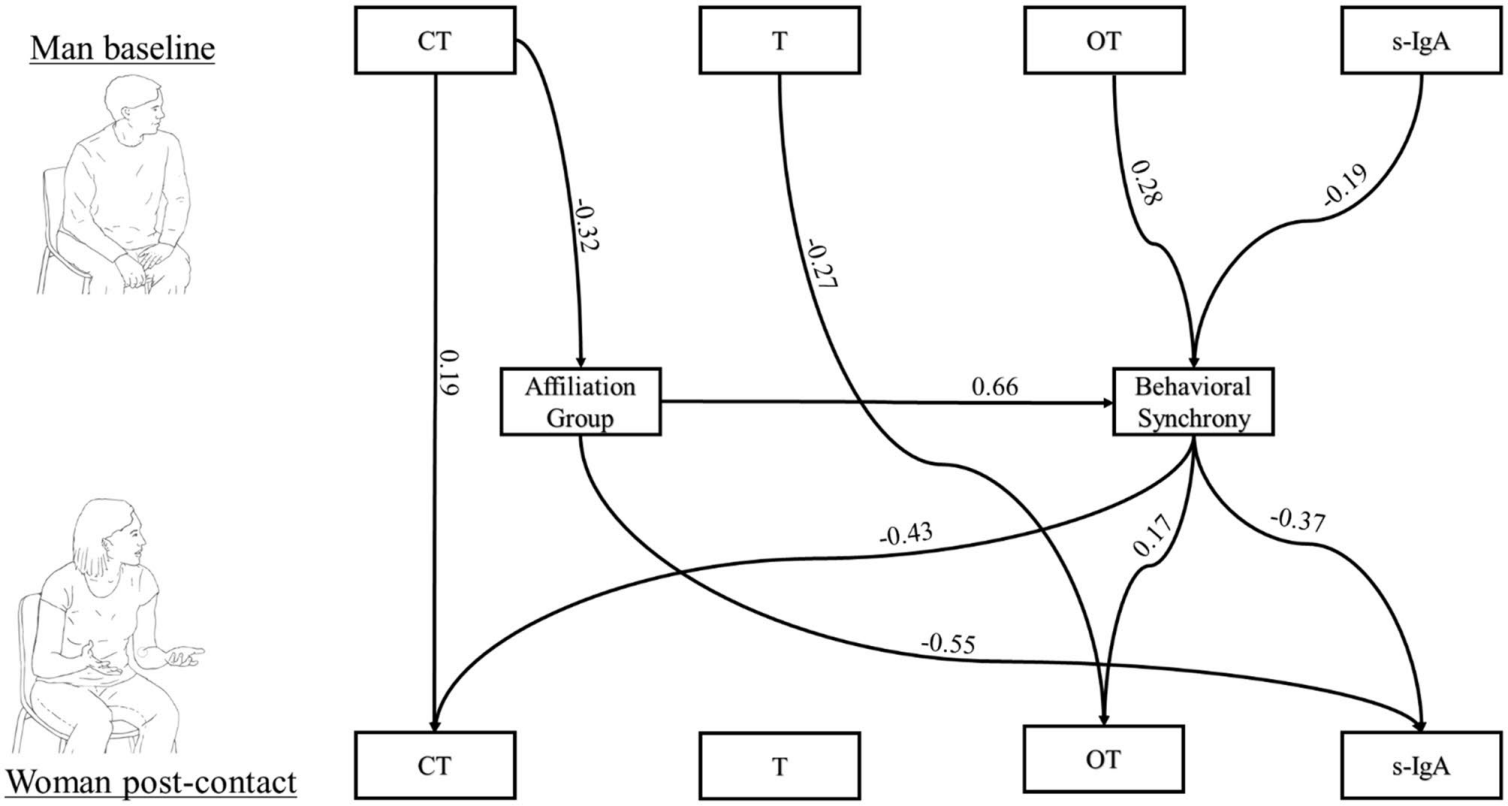
Path analysis for predicting women’s post-dialogue biomarkers values by men’s pre-dialogue values, mediated by group and behavioral synchrony. Significant paths are marked with a line and coefficient (*p* < 0.05). Affiliation group was coded as 0-Strangers, 1-Affilation. Women’s initial biomarkers were controlled by women’s baseline values. Paths were tested with bootstrap of 5,000 samples. Overall, the model provided an adequate fit to the data: χ^2^_(37)_ = 46.408, *p* = 0.138, CFI = 0.958, TLI = 0.928, RMSEA = 0.056.

The first model tested the associations between a man's baseline hormones and his female partner's hormonal response as mediated by *affiliation group* and *behavioral synchrony* (Fig. 2), while controlling for the woman's initial biomarkers. Direct paths were found between the man's baseline CT and the woman's CT at post-dialogue and between the man’s baseline T and the woman's post-dialogue OT. Several paths were mediated by affiliation group and behavioral synchrony. Affiliation group mediated the association between a man's baseline CT and the woman's post-dialogue s-IgA, with lower levels of CT and s-IgA associated with being within an attachment relationship. Test of mediation showed that this indirect path was significant (95% CI = 0.06, 0.335). The associations between the man's baseline CT and the woman's post-dialogue CT, OT, and s-IgA were mediated by affiliation group and behavioral synchrony. Men within an affiliative bond had lower CT and higher behavioral synchrony, which, in turn, linked with lower women's post-dialogue CT and s-IgA and higher OT levels (95% CI = 0.013, 0.096, 95% CI = − 0.075, − 0.002, and 95% CI = − 0.178, − 0.023, respectively). Additionally, behavioral synchrony mediated the relationship between the man's baseline OT and s-IgA and his female partner's CT, OT and s-IgA; higher baseline OT and lower s-IgA in men were associated with higher behavioral synchrony, which, in turn, linked with increased post-dialogue OT levels and decreased CT and s-IgA levels in their female partners (mediation tests were found significant, *p* < 0.05). Model fit was adequate (*χ*^*2*^_*(37)*_ = 46.408, *p* = 0.138, CFI = 0.958, TLI = 0.928, RMSEA = 0.056).

**Figure 3:**
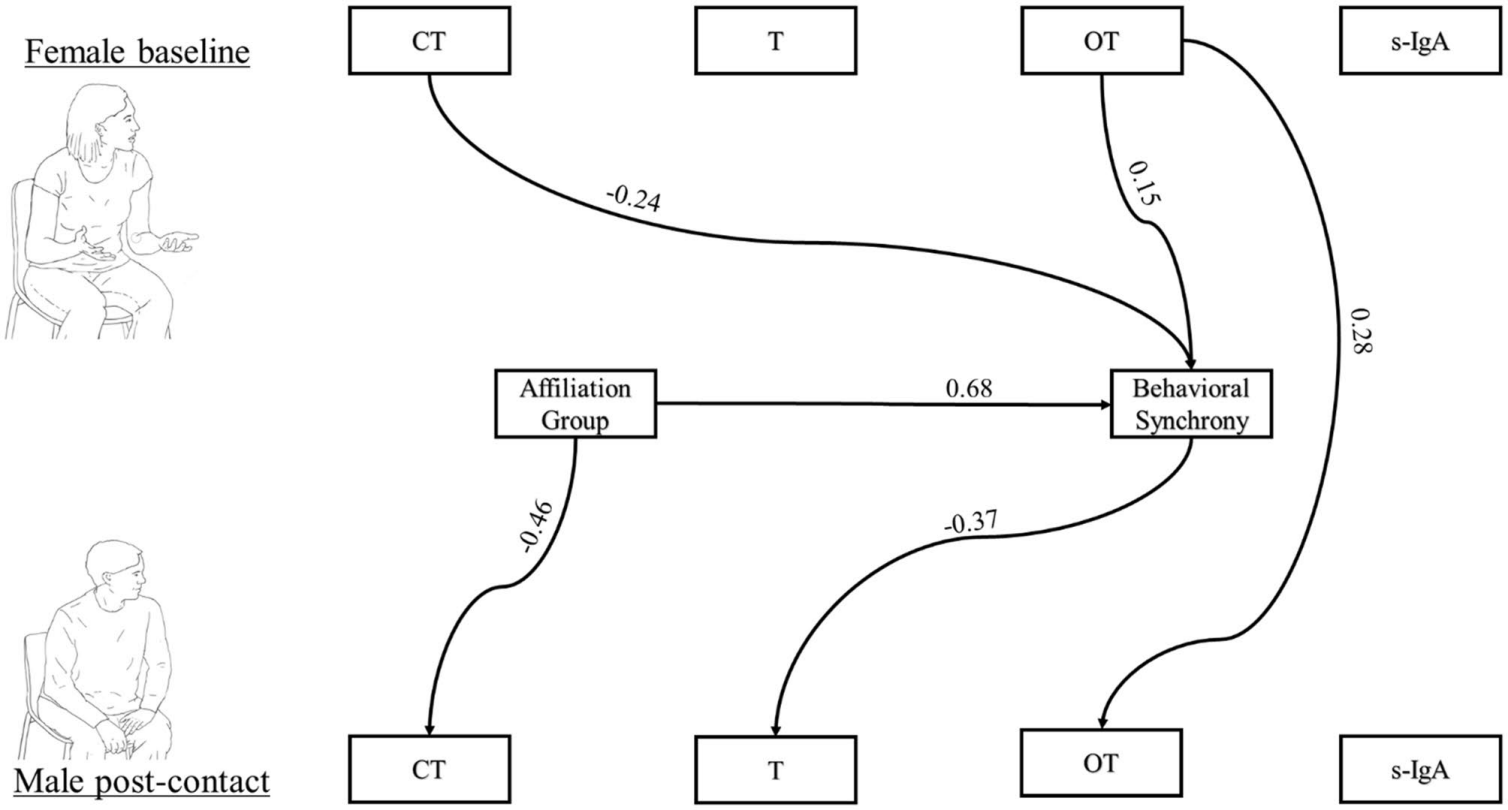
Path analysis for predicting men’s post-dialogue biomarkers values by women’s pre-dialogue values, mediated by group and behavioral synchrony. Significant paths are marked with a line and coefficient (*p* < 0.05). Affiliation group was coded as 0-Strangers, 1-Affilation. Men’s biomarkers were controlled by men’s baseline values. Paths were tested with bootstrap of 5,000 samples. Overall, the model provided an adequate fit to the data: χ2(25) = 28.238, *p* = 0.297, CFI = 0.983, TLI = 0.969, RMSEA = 0.04.

In the second model, we examined the associations between a woman's baseline hormones and her male partner's hormonal response as mediated by affiliation group and behavioral synchrony, controlling the man's initial biomarkers levels (Fig. 3). A direct path was found between the woman’s baseline OT and the man’s post-dialogue OT levels. Behavioral synchrony mediated the links between the woman's baseline OT and CT and the man's post-dialogue T levels. Lower levels of baseline CT and higher OT in the woman were associated with higher behavioral synchrony, which, in turn, linked with a decrease in her male partner's T at post-dialogue (for pre-CT- post T path 95% CI = 0.022, 0.144; pre-OT-post T 95% CI = − 0.105, − 0.013). Furthermore, behavioral synchrony mediated the link between affiliation group, with no significant pre-dialogue effects, and men's post-dialogue T levels (95% CI = − 0.678, − 0.169). Significant links were also found between affiliation group and behavioral synchrony and the man's post-dialogue CT levels. Model fit was adequate (χ^2^_(25)_ = 28.238, *p* = 0.297, CFI = 0.983, TLI = 0.969, RMSEA = 0.04).

## Discussion

Results of the current study indicate that social dialogue triggers a complex set of mutual influences between the partners' hormones that are sex-specific, shaped by the partners' affiliative status and behavioral attunement, and express differently in each endocrine system. Such mutual-influences are consistent with the formulations of the *bio-behavioral synchrony *model, which describes the coordination between the physiological and behavioral processes of social partners during moments of social contact^2,7^. Biobehavioral synchrony is theorized to have played an important role in the evolution of humans' social abilities by facilitating mindreading and communicative language and is thought to sustain human resilience, attachment, and the integration of individuals into social group^37^. Our data highlight several important findings that may shed further light on processes of biobehavioral synchrony across multiple endocrine systems as mediated by social coordination. First, we show that social dialogue between partners, whether within an affiliated bond or ingroup members, increases biomarkers of affiliation and anxiolytics (OT), decreases wear-and-tear and stress (CT), and reduces social comparison, competitiveness, and aggression (T). We further show that being within an attachment relationship with one's social partner impacts both social behavior and hormonal response; dialogue with a romantic partner or close friend was associated with greater behavioral synchrony and higher OT levels but lower CT, T and s-IgA levels. Additionally, our path analyses models describe the complex net of cross-sex cross-hormone influences partners exert on each other's endocrine response, both directly and via the mediating role of attuned social behavior. We found that the effects men exert on women's endocrine response differ from those women exert on men, underscoring the distinct risks and benefits embedded in social contact with the other sex for women and men. Overall, our findings indicate that while "relationship science" is gaining prominence as a framework for the study of growth and well-being^38^, attention to the biological layer of relationships may open new perspectives on the potential for resilience embedded in human attachments.

The main impact of social dialogue was observed for OT and CT, two key neuroendocrine systems originating in hypothalamic neurons that underpin bonding and enable mammals to manage stress through relationships^8,13^. Extant evidence in animal models underscored the modulatory effects of OT on HPA-axis activity^39^ and findings in humans similarly point to the role of close relationships in suppressing CT production^40^ and to the effects of OT on regulating stress reactivity in couples^33^. Yet, human studies have rarely studied peripheral levels of OT and CT together, and among the few that measured OT and CT in the context of attachment, OT's stress-modulating function was found to be more primitive as compared to CT, whose role in stress reactivity was more nuanced^41^. Here, we similarly found a significant increase in OT following social contact, similar to findings in other mammals^7^, highlighting the ubiquitous response of the OT system to naturalistic dialogue with members of one's social community. In contrast, while CT showed an overall decrease, this was defined by group and sex; men's CT decreased measurably after dialogue with a romantic partner, less so with a close friend, and none after encounter with a female stranger. This is consistent with perspectives suggesting that the beneficial effects of close relationships on health may stem, in part, from their impact on HPA-axis suppression^42^. Similarly, T levels showed an overall decline, but while the steepest decrease was observed for men interacting with their long-term romantic partner, those interacting with a strange female showed no attenuation. This is consistent with studies that showed a decrease in T levels in men within a committed relationship compared to single men in the context of available females^36,43^. These findings highlight the complex effects of dialogue on biomarkers of stress and competition in men and their dependence on the interactive context and the attachment relationship between the man and his female partner.

The models charting cross-hormonal influences between a man's initial hormonal profile and the woman's endocrine response and vice versa revealed several novel findings, particularly with regards to biomarkers of stress versus biomarkers of affiliation. With regards to the man's stress (CT) and social rank and competitiveness (T) biomarkers, we found that a man's baseline CT and T had a direct impact on the woman's stress and affiliative biomarkers at post-dialogue (CT and OT, respectively). In contrast, a woman's initial stress-related profile had no direct influence on the man's stress- and competitiveness-related biomarkers and impacted the man's CT and T only through the mediating role of behavioral synchrony (T) or by being within an attachment relationship (CT). This may suggest that women are more sensitive to a man's biological state, particularly to signs of stress, aggression, competitiveness, and social status, findings which are consistent with research in other primates. For instance, while female marmosets' behavior during social contact is related to males’ baseline CT levels, the opposite is not observed for males^44^. Similarly, glucocorticoid levels and grooming behavior of wild female baboons link with instability in the alpha male's rank position^45^, and in chimpanzees, reduced urinary glucocorticoids levels were found in females after time spent with their male partners, but not vice versa^46^. Our findings similarly underscore the direct effects of a human male's stress and status biomarkers on his female partner's multi-system endocrine response.

A somewhat mirror sex-related result emerged in relation to OT, the affiliation biomarker. Here, a woman's baseline OT had a direct impact on the man's OT at post-dialogue regardless of behavioral style or attachment status. In contrast, a man's baseline OT had no direct impact on the woman's hormones and linked with her post-dialogue OT, CT, and s-IgA only through the mediating role of behavioral synchrony. This suggests that men may be more sensitive to signs of women's affiliation and attachment, consistent with research in biparental species indicating that fatherhood-related OT increases in a male animal are shaped by the elevation of OT in his female partner during pregnancy and birth^47^. These results extend previous studies on the mutual influences of OT between attachment partners, including mother–child, father-child, and new-lovers pairs, which were both direct and mediated by synchronous behavior^16,48^. Here we show that such cross-person effects of OT are observed in all partners within one's social group following a social dialogue: long-term romantic couples, friends, and ingroup community members. These findings may suggest that when two humans who come from the same cultural community interact with respect and mutuality, the dialogue has the potential to enhance the partner's OT levels, triggering OT's beneficial effects on sociality, anxiolytics, and immune-system enhancement^49^.

With regards to T, a biomarker of competitiveness, assertiveness, and social-rank focus in both humans and other primates^50^, our findings indicate that a man's high initial T had a direct impact on reducing the woman's post-dialogue OT levels and that this direct effect was not modulated by behavioral synchrony or attachment. This suggests that men's high initial T may be a biomarker of traits that are less amenable to change through social interactions and may function to suppress biomarkers of affiliation, calm, and connectedness in his female partner. In comparison, a woman's initial T levels, typically linked with behavioral hostility and changes in moral judgment^16,51,52^, had no direct or mediated effects on the man's endocrine response in any system. Of note, men's T at post-dialogue were attenuated if the dialogue was synchronous, suggesting that aggression and competitiveness in men can be fine-tuned through a mutual and well-matched dialogue. Similarly, we found that s-IgA, a biomarker of the immune system that increases during periods of stress and its decrease is associated with greater well-being^53,54^, could be attenuated in women, but not in men, at post-dialogue via the mediating role of affiliation and behavioral synchrony, highlighting the greater sensitivity of women's immune system to a mutual and empathic dialogue.

The mediated paths through social behavior suggest that the degree of mutual influences between the partners' hormones may be stronger among affiliated partners, as behavioral synchrony was elevated in these pairs. Mutual influences in relation to CT as moderated by attachment and behavior may be of interest in this context. While a man CT could have a modulatory effect on the woman's stress response both directly and through the mediating path of attachment and synchrony, there were no direct paths by which a woman could impact her male partner's post-dialogue CT levels, apart from the mediated effects of attachment. This, again, highlights the greater sensitivity of women to their male partners' stress levels, which likely had survival consequences across the evolution of *Homo sapiens*. In contrast, women's, but not men's CT directly impacted the level of behavioral synchrony between the partners and this finding indicates that the social atmosphere is shaped to a greater extent by the woman's physiological stress and anxiety.

Finally, it is important to emphasize that our results describe correlations and by no mean imply causality, and the term "effects", "impact", and "influences" used here describes statistical, not causal effects. Limitations of the study relate to the omission of same-sex social partners and the mutual influences they exert on each other's biology and behavior, in both couples, close friends, and strangers, and such research should be a natural next step. Furthermore, while our study tapped the most central neuroendocrine systems of sociality across mammalian species as well as a reliable salivary biomarker of immunity, there are other systems that could be studied in relation to social dialogue (e.g., endorphins).

Since the discovery of social bonding by Lorenz, the early work of the ethologists, and the incorporation of their findings into Bowlby's attachment theory^4^, it has become clear that the human brain, like that of other mammals, is shaped through the mutual influences of physiological, sensory, and behavioral signals between mother and young. These experiences program the infant's brain and behavior for life within affiliative bonds and social groups. It has been further suggested that romantic attachments and close friendships are sustained by the same biobehavioral mechanisms as those underpinning the mother-infant bond and that attachment relationships throughout life provide the foundation for resilience and well-being^2^. Our findings describe how social dialogue between human adults enhances well-being through cross-hormone cross-person biobehavioral mechanisms spanning a broad-band neuroendocrine response. By doing so, our findings contribute to knowledge on the effects of social contact on well-being. Our results may add to the emerging research on the neurobiology of affiliation, a new field of research that aims to formulate more comprehensive models on how human relationships build brain, enhance well-being, and sustain resilience.

## Methods

### Procedure

Participants were recruited through ads posted at a university campus and its surrounding areas and via internet forums. Before arrival, participants completed self-report questionnaires considering demographic and health information and depression (Beck Depression Inventory; BDI). Once participants arrived to the laboratory, they were seated next to each other with a dividing screen between them and were guided not to talk in order to maintain unfamiliarity (for strangers) and keep fixed conditions between groups. Next, an explanation about the experiment and the paradigms ahead was given, participants signed informed consent, gave their first saliva sample, and electroencephalogram caps were placed by trained experimenters.

Participants engaged in three naturalistic interactions consistent with our prior research^15,16,30^. Altogether, the social dialogue lasted for 20 min, which is sufficient to elicit a hormonal response. (1) *Positive interaction—*participants were asked to plan "the best day ever" to spend together. (2) *Empathy giving*– participants were asked to share a distressing personal event unrelated to the partner. After 5 min, the experimenter asked participants to reverse roles. (3) *Conflict—*before the interaction began partners were given a list of topics that typically elicit conflict among social partners and were asked to choose a topic that is conflictual in their relationship. Strangers chose from the same list topics they feel strongly about in their current or future relationships.

### Participants

The study included 164 young adults in male–female pairs (N = 82 pairs) recruited in three affiliations groups (1) *Couples—*partners within a committed romantic relationship and cohabitation of at least 1 year (time together: M = 3.83, SD = 2.85 years), (2) *Friends—*Consistent with Dunbar^12^, best friends were those who considered each other among their top five “best friends”, were never involved romantically, and their period of familiarity was comparable to that of the couple's (time of close friendship: M = 4.10, SD = 2.39, no different than couples’, t_(38)_ = − 0.31, *p* = 0.76), in order to tease apart the effects of familiarity from those of romantic love and cohabitation (3) *Strangers—*demographically-matched male and female from the same in-group who met for the first time during the experiment. Exclusion criteria included medication intake, physical or psychiatric condition, and self-reported health problems (such as asthma, blood pressure, head injuries, etc.). No significant differences were found in demographic variables such as age, education, or indices of depression (see Table 1).

### Social behavior coding

We used Coding Interactive Behavior manual (CIB), a well-validated global rating system for coding social interactions. The CIB has been validated across ages, cultures, and risk conditions (for review^13^), and the Adult CIB manual was used here, which utilizes multiple codes integrated into theoretically-based constructs^16^. The synchrony construct of the CIB was used; this construct comprises codes related to reciprocity, inclusion, mutual involvement, initiation of social bids, creativity, fluency, and joint expansion of dialogue. Each of the four session was coded separately (empathy giving was coded separately for each partner) and the synchrony construct from the four paradigms was averaged into a *Behavior Synchrony* construct (Cronbach's α = 0.86). Coding was conducted by trained coders and blind to any other information. Reliability was conducted on 15% of the interaction with reliability exceeding 95% (intraclass r = 0.93).

### Hormone collection and determination

#### Saliva samples collection and preparing for analysis

Saliva samples were collected by passive drooling into clean 5 ml tube and stored at − 20 °C. To precipitate the mucus, samples underwent three freeze–thaw cycles, freeze at − 70 °C and thaw at 4 °C. After the fourth cycle the tubes were centrifuged twice at 1500 × g (4000 rpm) for 20 min. Supernatant was collected and the aliquots stored at − 20 °C until assayed.

In order to increase the sensitivity OT Enzo-EIA kit the liquid samples were first freeze-dried for 3–4 days to yield a cotton-like powder. Prior the freeze-drying procedure the samples were stored at − 80 °C, for at least three days. The dry powder was kept at − 20 °C until assayed. Second, the dry samples were reconstructed with the assay buffer, in forth of the original volume, immediately before the assay by the ELISA Kit.

#### Measuring the markers by ELISA method

Salivary concentrations of the four hormones were measured using commercial Linked Immunosorbent Assay (ELISA) kits: Oxytocin by ENZO (New-York, USA), CT and T by Salimetrics (Pennsylvania USA), and s-IgA by Euroimmun (Lubeck Germany) according to the kit's instructions and consistent with prior research^55,56^. Each kit provides a quantitative in vitro assay for the biomarker in human saliva. Measurements were done in duplicate according to the instructions recommended by the respective manufacturer. The concentration of each hormone in the sample was calculated by MEGELAN (Tecan, Germany) according to relevant standard curves. The intra-assay and inter-assay coefficients of samples as measured by the manufacturer's control are as follows: for OT less than 9.7% and 14.5%, for CT less than 4.7% and 9.3%, for T less than 5.97% and 12.1%, and for s-IgA less than 4.1% and 4%.

### Statistical analysis

Due to different scales between hormones and sexes, we used the z-score transformation of the values within each hormone and between sexes. Analysis of variance (ANOVA) and mixed models were used to assess our first hypothesis on mean-level changes in each hormone following social dialogue in women and men according to affiliation status. Pearson correlations examined associations between variables. To test direct and mediated effects of one partner's baseline hormonal profile on the other partner's hormonal reactivity as mediated by attachment status and behavioral synchrony, path analyses were used separately for women and men. Path analysis was based on maximum likelihood estimations and indicators of model fit were: χ^2^, comparative fit index (CFI), Tucker–Lewis Index (TLI), and root mean square error of approximation (RMSEA). Model fit for χ^2^ statistic is expected to be nonsignificant in the case of adequate fit, CFI and TLI equal to or greater than 0.90, RMSEA equal to or less than 0.06 are indicative of adequate fit to the data^57^. Significance of the mediation effects was assessed using a procedure recommended by Hayes (2013) and calculated the 95% confidence intervals of 5,000 bias-corrected and bootstrapped analyses^58,59^. In cases where the value zero is not included in the confidence interval, this indicates significant effect at α < 0.05. All statistical analyses were done using in R 3.5.3^60^.

### Ethical considerations

Study was approved by the Institutional Review Board (IRB) of Bar-Ilan University. All procedures were explained to the participants before the study and were performed in accordance with ethical guidelines. Participants gave written informed consent and were free to leave the experiment at any time with full compensation. Participants received 50 USD for participation.

## Supplementary Information

Supplementary Information.

## Data Availability

The data that support the findings of this study are available on request from the corresponding author, R.F. The data are not publicly available due to its nature*—*questionnaires and videos of interactions containing information that could compromise the privacy of research participants.

## Abbreviations

IRB: Institutional Review Board
CIB: Interactive behavior manual
ANOVA: Analysis of variance
CFI: Comparative fit index
TLI: Tucker–Lewis index
RMSEA: Root mean square error of approximation
