# Supplementary material for: Social dialogue triggers biobehavioral synchrony of partners' endocrine response via sex-specific, hormone-specific, attachment-specific mechanisms
Source: Sci Rep. 2021 Jun 14;11:12421. doi: 10.1038/s41598-021-91626-0 (PMC8203689; doi:10.1038/s41598-021-91626-0)
Supplement: Supplementary file 1 — Supplementary Information. [file 41598_2021_91626_MOESM1_ESM.pdf]

Social Dialogue Triggers Biobehavioral Synchrony of Partners' Endocrine Response via Sex-Specific, Hormone-Specific, Attachment-Specific Mechanisms

Amir Djalovski[1, 2]

Sivan Kinreich[3]

Orna Zagoory-Sharon[1]

Ruth Feldman[1, 4]

[1]Center for Developmental Social Neuroscience, Interdisciplinary Center Herzliya, Herzliya, Israel

[2]Department of Psychology, Bar-Ilan University, Ramat Gan, Israel

[3]Department of Psychiatry, State University of New York Downstate Medical Center, Brooklyn, NY, USA

[4]Child Study Center, Yale University, New Haven, CT

Corresponding author:

Ruth Feldman, PhD

Center for Social Neuroscience

Interdisciplinary Center, Herzliya, Israel

Email: feldman.ruth@gmail.com

Supplementary material

Table S1 – correlation matrix with 95% confidence interval for pre- post-dialogue hormonal levels

Variable	1	2	3	4	5	6	7	8	9	10	11	12	13	14	15
1. CT Female Pre															
2. CT Female Post	0.17<br>[-.05, .38]														
3. CT Male Pre	0.15<br>[-.06, .36]	0.31<br>[.10, .50]													
4. CT Male Post	-0.13<br>[-.34, .09]	0.17<br>[-.04, .38]	0.28<br>[.07, .47]												
5. OT Female Pre	-0.06<br>[-.28, .16]	0.18<br>[-.03, .39]	0.15<br>[-.07, .35]	-0.1<br>[-.31, .12]											
6. OT Female Post	0.03<br>[-.19, .24]	-0.11<br>[-.32, .11]	-0.04<br>[-.25, .18]	-0.05<br>[-.26, .17]	0.52<br>[.35, .66]										
7. OT Male Pre	-0.04<br>[-.26, .17]	-0.01<br>[-.23, .21]	0.14<br>[-.07, .35]	0.08<br>[-.14, .29]	-0.05<br>[-.26, .17]	0.04<br>[-.18, .25]									
8. OT Male Post	-0.02<br>[-.24, .20]	0<br>[-.22, .22]	0.04<br>[-.18, .25]	-0.02<br>[-.24, .20]	0.26<br>[.04, .45]	0.03<br>[-.19, .25]	0.51<br>[.33, .65]								
9. SIGA Female Pre	-0.27<br>[-.46, -.06]	0.19<br>[-.03, .39]	0.04<br>[-.18, .26]	0.26<br>[.05, .46]	0.16<br>[-.06, .36]	0.11<br>[-.11, .32]	0.1<br>[-.12, .31]	-0.01<br>[-.23, .21]							
10. SIGA Female Post	-0.16<br>[-.36, .06]	0.09<br>[-.13, .30]	0.05<br>[-.17, .26]	0.26<br>[.04, .45]	0.13<br>[-.09, .33]	0.08<br>[-.14, .29]	0<br>[-.22, .21]	-0.1<br>[-.31, .12]	0.24<br>[.03, .44]						
11. SIGA Male Pre	-0.04<br>[-.25, .18]	0.06<br>[-.16, .27]	0<br>[-.22, .22]	0.02<br>[-.20, .23]	-0.21<br>[-.41, .01]	-0.22<br>[-.42, -.00]	0.02<br>[-.20, .24]	-0.01<br>[-.23, .21]	0.1<br>[-.12, .31]	-0.11<br>[-.32, .11]					
12. SIGA Male Post	-0.06<br>[-.27, .16]	-0.19<br>[-.39, .03]	0.09<br>[-.13, .30]	0.23<br>[.02, .43]	0.02<br>[-.20, .23]	0.01<br>[-.20, .23]	0.03<br>[-.19, .25]	0.08<br>[-.14, .29]	0.29<br>[.08, .48]	0.22<br>[-.00, .41]	0.11<br>[-.11, .32]				
13. TS Female Pre	0.26<br>[.05, .45]	0.17<br>[-.05, .37]	0.27<br>[.05, .46]	-0.07<br>[-.29, .15]	0.2<br>[-.02, .40]	0.06<br>[-.16, .27]	0.23<br>[.02, .43]	0.16<br>[-.05, .37]	-0.1<br>[-.31, .12]	0.22<br>[.00, .42]	0.05<br>[-.17, .26]	0.09<br>[-.13, .30]			
14. TS Female Post	0.23<br>[.02, .43]	0.15<br>[-.06, .36]	0.3<br>[.09, .48]	-0.19<br>[-.39, .03]	0.23<br>[.01, .42]	0.1<br>[-.12, .31]	0.09<br>[-.13, .30]	0.09<br>[-.13, .30]	-0.17<br>[-.37, .05]	0.08<br>[-.13, .30]	-0.01<br>[-.23, .21]	-0.06<br>[-.27, .16]	0.6<br>[.44, .72]		
15. TS Male Pre	-0.14<br>[-.35, .08]	0.05<br>[-.16, .27]	0.26<br>[.04, .45]	0.25<br>[.04, .45]	-0.01<br>[-.23, .20]	-0.28<br>[-.47, -.07]	-0.06<br>[-.27, .16]	0.06<br>[-.16, .27]	0<br>[-.22, .22]	-0.11<br>[-.32, .11]	0.09<br>[-.13, .30]	0.03<br>[-.19, .25]	-0.12<br>[-.33, .10]	-0.06<br>[-.28, .16]	
16. TS Male Post	-0.05<br>[-.26, .17]	0.18<br>[-.03, .39]	0.23<br>[.01, .43]	0.49<br>[.30, .64]	-0.21<br>[-.41, .01]	-0.35<br>[-.53, -.15]	0.01<br>[-.21, .23]	0.01<br>[-.21, .23]	0.11<br>[-.11, .32]	0.02<br>[-.20, .23]	-0.02<br>[-.24, .20]	0.23<br>[.01, .42]	-0.11<br>[-.32, .11]	-0.08<br>[-.29, .14]	0.38<br>[.17, .55]

Note: Values in square brackets indicate the 95% confidence interval for each correlation.
